# *In Silico* Insights into the Symbiotic Nitrogen Fixation in *Sinorhizobium meliloti* via Metabolic Reconstruction

**DOI:** 10.1371/journal.pone.0031287

**Published:** 2012-02-01

**Authors:** Hansheng Zhao, Mao Li, Kechi Fang, Wenfeng Chen, Jing Wang

**Affiliations:** 1 State Key Laboratory of Agrobiotechnology, College of Biological Sciences, China Agricultural University, Beijing, People's Republic of China; 2 Key Laboratory of Mental Health, Institute of Psychology, Chinese Academy of Sciences, Beijing, People's Republic of China; Universidad Nacional Autonoma de Mexico, Instituto de Biotecnologia, Mexico

## Abstract

**Background:**

*Sinorhizobium meliloti* is a soil bacterium, known for its capability to establish symbiotic nitrogen fixation (SNF) with leguminous plants such as alfalfa. *S. meliloti* 1021 is the most extensively studied strain to understand the mechanism of SNF and further to study the legume-microbe interaction. In order to provide insight into the metabolic characteristics underlying the SNF mechanism of *S. meliloti* 1021, there is an increasing demand to reconstruct a metabolic network for the stage of SNF in *S. meliloti* 1021.

**Results:**

Through an iterative reconstruction process, a metabolic network during the stage of SNF in *S. meliloti* 1021 was presented, named as *i*HZ565, which accounts for 565 genes, 503 internal reactions, and 522 metabolites. Subjected to a novelly defined objective function, the *in silico* predicted flux distribution was highly consistent with the *in vivo* evidences reported previously, which proves the robustness of the model. Based on the model, refinement of genome annotation of *S. meliloti* 1021 was performed and 15 genes were re-annotated properly. There were 19.8% (112) of the 565 metabolic genes included in *i*HZ565 predicted to be essential for efficient SNF in bacteroids under the *in silico* microaerobic and nutrient sharing condition.

**Conclusions:**

As the first metabolic network during the stage of SNF in *S. meliloti* 1021, the manually curated model *i*HZ565 provides an overview of the major metabolic properties of the SNF bioprocess in *S. meliloti* 1021. The predicted SNF-required essential genes will facilitate understanding of the key functions in SNF and help identify key genes and design experiments for further validation. The model *i*HZ565 can be used as a knowledge-based framework for better understanding the symbiotic relationship between rhizobia and legumes, ultimately, uncovering the mechanism of nitrogen fixation in bacteroids and providing new strategies to efficiently improve biological nitrogen fixation.

## Introduction

*Sinorhizobium meliloti* is a model bacterium belonging to rhizobia, known for its capability to perform symbiotic nitrogen fixation (SNF) within leguminous host plants (mainly in the genera of *Medicago*, *Melilotus*, and *Trigonella*). It thereby has gained a widespread interest for the potential benefits to sustainable agriculture and ecosystem, as well as to biological discoveries. SNF is an alternative way to substitute traditional industrial production of nitrogenous fertilizer, which consumes energy and causes serious environmental pollution. As previously report, SNF has accounted for a quarter of the nitrogen fixed annually on earth [1]. Prior to performing SNF function by rhizobia, additional two developmental stages are necessary. Firstly, a complex signal exchanges between the symbiotic partners triggers the invasion of the plant roots by rhizobia. Then, rhizobial bacteria induce the plant roots to form a special structure, called root nodule, within which *S. meliloti* cells penetrate into the plant cells in the root context through formation of infection tread. Once the bacteria are engulfed within host cell membranes, the bacteria differentiate into nitrogen-fixing bacteroids [2]. In the stage of SNF, there is a tight metabolic association between the host plants and rhizobial bacteria. In general, plants supply rhizobial bacteria with dicarboxylic acids as carbon and energy sources [3], in return, rhizobial bacteria provide the legume usable nitrogen in the form of ammonium, using their inherent ability to reduce atmosphere nitrogen gas. This process is accompanied by the cycling of amino acids between the partners [4], [5].

However, SNF is too complex to be figured out by experimental methods alone. Many factors involved in SNF remain unknown. With the completion of whole genome sequencing of *Sinorhizobium meliloti* strain 1021, which consists of a chromosome of size 3.65 Mb and two megaplasmids, pSymA and pSymB, of 1.36 Mb and 1.68 Mb, respectively [6]–[9], as well as the advent of various high-throughput experimental data for *S. meliloti* [10]–[16], it is possible and necessary to systemically analyse its metabolic capabilities and to search for key genes required for SNF in the manner of *in silico* metabolic reconstruction. Model *i*OR363 is a previously published metabolic reconstruction for *Rhizobium etli* CFN42 [17], which is also a nitrogen fixing bacterium but with different physiological features from *S. meliloti* in many aspects. For instance, *R. etli* induces its host plants such as beans (*Phaseolus vulgaris*) to develop determinate nodules. In contrast, *S. meliloti* induces its legume hosts to form indeterminate nodules. These differences lead to a variety of different underlying metabolic properties between *S. meliloti* and *R. etli.*

In this study, we presented a manually curated metabolic network for the stage of SNF in *S. meliloti* 1021, named as *i*HZ565, which accounts for the main metabolic pathways participated in the stage of SNF. A novelly defined objective function (OF) was formulated and applied to the model-driven analysis and discoveries, including refinement of gene annotation and prediction of SNF essential genes, that are indispensable for efficient SNF in bacteroids. As the first metabolic network for the stage of SNF in *S. meliloti* 1021, model *i*HZ565 provides a biochemically and genomically structured knowledgebase to explore the metabolic properties of SNF from a systematic perspective [18], and is a foundation for predictive phenotype modeling, being associated with the simulation approach of flux balance analysis (FBA) [19], [20]. It allows a broad spectrum of basic and practical applications, especially in the mechanism of nitrogen fixation in bacteroids and the new strategies to improve biological nitrogen fixation efficiently.

## Results and Discussion

### Reconstruction of metabolic network involved in symbiotic nitrogen fixation in *S. meliloti* 1021

**Figure 1:**
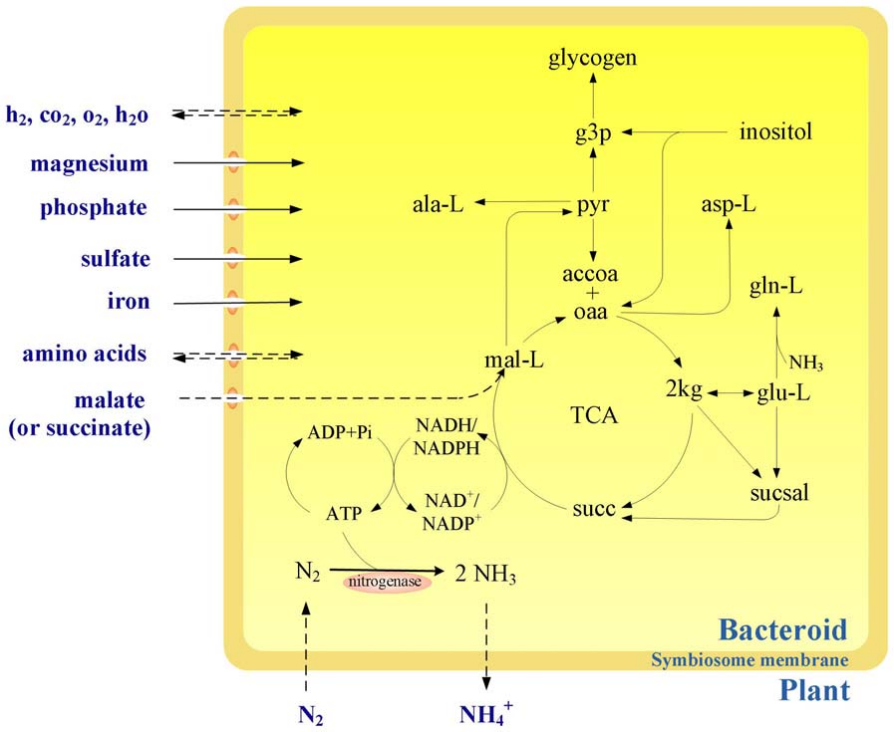
Schematic representation of the metabolic network during the stage of SNF in *S. meliloti* 1021.

The reconstructed symbiotic nitrogen-fixing metabolic model of *S. meliloti* 1021, termed *i*HZ565, following the conventional naming rules [18], includes 503 metabolic and transport reactions, 522 metabolites, and 565 genes, covering nearly 10% of the 6,218 protein coding genes identified from whole genome sequencing in Table 1. The main metabolic pathways involved in the stage of SNF (*i.e.* after the stages of infecting plant roots and developing nodules inside the plant) were reconstructed in *i*HZ565. The overall reconstructed metabolic network is available in the File S1 and the model in SBML format is in the File S2. The global SNF metabolic network indicates that the nitrogen-fixing process is tightly coupled with the nutrient sharing system between *S. meliloti* and host plants, the carbon and energy metabolism, as well as some crucial cofactors required for SNF in Figure 1.

**Table 1:** Comparison of the reconstructed metabolic networks during the SNF stage for *S. meliloti* 1021 (*i*HZ565) and *R. etli* CFN42 (*i*OR363).

Model	*i*HZ565	*i*OR363
Genome size	6.69 Mb	6.53 Mb
Included genes	565	363
Total reactions	503	387
Gene-associated reactions(% of total reactions)	481(92.4%)	318(82.2%)
Non-gene-associated reactions	15	63
Spontaneous	7	6
Exchange reaction	23	12
Metabolites	522	371

**Figure 2:**
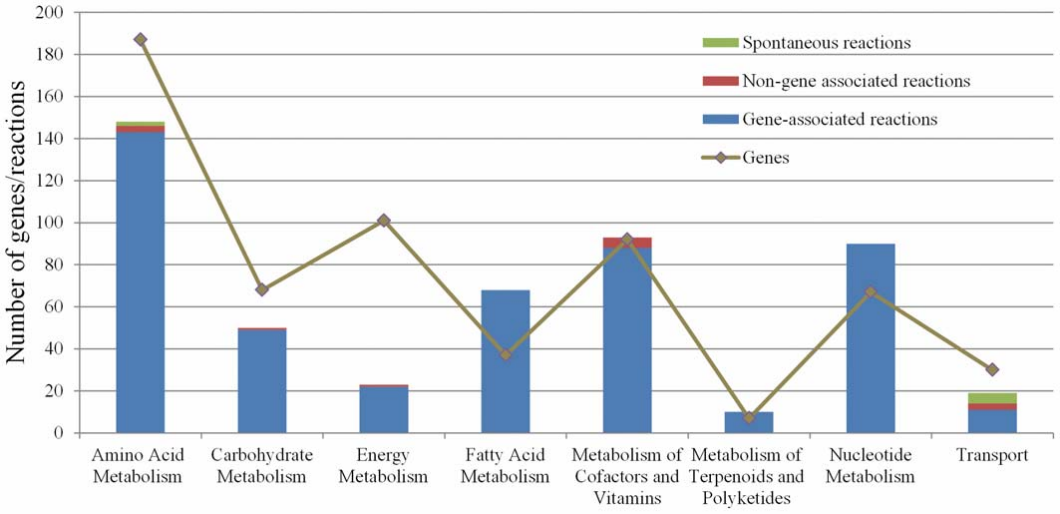
The distribution of genes and gene-associated, non-gene-associated, or spontaneous reactions on each pathway of model *i*HZ565.

The distribution of genes, gene-associated and non-gene-associated reactions on each metabolic pathway of *i*HZ565 was illustrated in Figure 2. The subsystem of amino acid metabolism contains the largest number of reactions. The experimental results demonstrate that *S. meliloti* 1021 has the capacity to synthesize several amino acids [14]. Another large subsystem, namely the metabolism of cofactors and vitamins, includes essential components required by nitrogenase, which is a core enzyme in SNF and catalyses the ATP-dependent reduction of dinitrogen (N_2_) to ammonia (NH_3_). In the subsystem of energy metabolism, the number of genes is more than the number of reactions since most of these genes have multiple copies in order to meet the high demand for ATP consumed in the SNF.

### Formulating an SNF objective function

A critical step in the computational simulation and analysis of underdetermined metabolic systems is to set an appropriate objective function (OF) that can depict the specific biological process accurately. In general, a common assumption is that the cell aims to maximize its growth rate (*i.e.* biomass) or ATP production [21], [22]. However, in the stage of SNF, the bacteroids stop growing and only accomplish the single function, nitrogen fixation, acting as an organelle [23]. The practical optimal status in bacteroids has not been understood very well, and the particular challenge is how to carry out relevant experiments to uncover this. An effort made here was to give a suitable OF formulation that is distinct from traditional ones in order to accurately mimic the real SNF condition.

The definition of OF in *i*HZ565 was different from the previous one in *i*OR363, in which the OF only concerned about the function of nutrient sharing [17]. In our study, the defined OF was based on the assumption that two continuous biological processes, namely nutrient sharing between plant and bacteroids (Reaction 1) and the nitrogen fixation in bacteroids (Reaction 2), should be tightly coupled with each other. Another consideration is that the ammonia exported to plant should come from nitrogenase-catalysed reaction rather than other non-nitrogen fixation reaction indirectly generated.

**Reaction 1:**

**Alanine[c] + Aspartate[c] + Arginine[c] + Glycogen[c] + Hexadecanoate[c] + Ammonia[c] + (0.01) Co-factors (nitrogenase)[c] → Alanine[e] + Aspartate[e] + Ammonia[e]**

**Reaction 2:**

**(16) ATP + (6) Ferredoxin (reduced) + (16) Water + Nitrogen → (16) ADP + (6) Ferredoxin (oxidized) + (8) Hydrogen + (2) Ammonia + (16) Phosphate**

Herein, Reaction 1 is similar to a transport reaction, in which the symbols [c] and [e] represent that each metabolite is located in cytosol or extracellular compartment, respectively. Reaction 1 depicts the nutrient sharing process between bacteroids and their host plants in the stage of SNF, during which three metabolites (Alanine, Aspartate, and Ammonia) are generated in bacteroids, and subsequently export to the host plants. As for Arginine, Glycogen, Hexadecanoate, and Co-factors, they are synthesized and accummulated in bacteroids. During nitrogen fixation, Glycogen and Hexadecanoate serve as major carbon and energy storage, and Co-factors and Arginine are prerequisite to ensure that the nitrogenase functions properly. Reaction 2 is a common nitrogen-fixing biochemical reaction occurring in cytosol and catalysed by the enzyme — nitrogenase, which can reduce Nitrogen to Ammonia by six electrons, associated with the reduction of 2H^+^ to Hydrogen using the hydrolysis of 16 molecules of ATP in the cytoplasm. Reaction 1 and Reaction 2 stand for two biological processes with multiple interplays. In order to balance these two biological processes and simultaneously optimize nitrogen fixation rate in the stage of SNF, we set both Reaction 1 and Reaction 2 as the OF in *i*HZ565. The detailed explanation for the OF is as follows.

Amino acid cycling is an important part of nutrient sharing system in the stage of SNF. Aspartate and Alanine are generated in bacteroids and exported to the host plants when the host plants provide *S. meliloti* bacteroids with Glutamate. The amino acid exchange system may minimize the NH_4_^+^ assimilation of bacteroids, and promote the NH_4_^+^ exportation for plant growth [24], [25]. This kind of amino acid cycling has been validated to exist in *Rhizobium leguminosarum –* legume symbiosis [3], [4]. In addition, proteins for the relevant amino acids transport in *R. leguminosarum* are homologous with proteins of *S. meliloti*. It is proposed that these two species share a similar amino acid exchange system [14].

Another important amino acid is Arginine, whose metabolism is vital for generating ATP and membrane potential in the microaerobic condition of symbiosis [7]. Additionally, the enzyme for the Arginine biosynthesis was observed in the proteomic analysis of *S. meliloti* [14]. This is corresponding to the high concentration of polyamines, which use arginine as a precursor in root nodule of some legumes [26]. The role of polyamines in nutrient exchange between bacteroids and plants was investigated in soybean root nodules [27]. Therefore, the biosynthesis of Arginine in *Rhizobium* may be important for maintaining the symbiosis.

Ammonia (NH_4_^+^) is the focal point in this study as it plays a key role in the legume-rhizobia symbiosis. Through the production of nitrogenase complex, the endocelluar symbionts convert atmospheric nitrogen to NH_3_/NH_4_^+^ using Reaction 2 as utilizable form of nitrogen exported to the host plant.

It has been experimentally demonstrated that the *S. meliloti* mutant defective in synthesis of glycogen and fatty acid produced a less effective nitrogen fixation on *Medicago truncatula* and older nodules of *Medicago sativa* [28]. Thereby, addition of glycogen and fatty acid in the new OF would provide a better understanding for the role of the compounds in the overall metabolism of symbiotic nitrogen fixation.

The term Co-factors stands for the combination of a few important cofactors, which maintain basic metabolism and have been detected in proteomic assays (such as pyridoxine, adenosylcobalamin, thiamine, and glutathione) and participate in the synthesis of nitrogenase (such as heme, FeS cluster, molybdenum, and homocitrate). In particular, the typical molybdenum nitrogenase is comprised of two component proteins, namely the iron (Fe) protein and the molybdenum-iron (MoFe) protein. FeS cluster, molybdenum, and homocitrate are essential components of the two proteins [29], [30]. Rhizobia uptake Mo in the form of molybdate [31]. The *modABC* genes encoding molybdate transporter have been identified in *S. meliloti* genome [6]. Most rhizobia including *S. meliloti* do not possess *nifV* gene encoding homocitrate synthase, so rhizobia have to obtain homocitrate from host plants to synthesize nitrogenase [32]. Heme is synthesized by the bacterial symbiont and is an essential prosthetic group of leghemoglobin, which contributes to the establishment of a microaerobic environment in the nodule. Thus, it can support the respiration of bacteroids and is crucial for effective symbiotic nitrogen fixation [33], [34].

Since OF plays a crucial role in computational simulation and prediction via FBA, the two different OFs defined in *i*HZ565 and *i*OR363 were compared, and how OF affects the *in silico* results were evaluated. First, simulation results of *i*HZ565 were acquired by using the original OF defined in *i*HZ565. The *in silico* and *in vivo* results agreed with each other as shown in Table 2 and further illustrated in the next section. Second, the OF in *i*HZ565 was replaced by the OF in *i*OR363 with deletion of the compound of PHB, considering the inability of *S. meliloti* to produce PHB in bacteroids [28], to perform FBA simulation of *i*HZ565. Results of incomplete utilization of TCA and inactive pathways, including pentose phosphate pathway, sulfur, porphyrin, and chlorophyll metabolism, were obtained, which are against with experimental evidences [8], [33], [35]–[37]. This phenomenon is mainly due to the OF in *i*HZ565 has taken into account the component of “SNF-cofactor”, which is essential for the biosynthesis of nitrogenase, whereas the OF in *i*OR363 not.

**Table 2:** The activities of main pathways in the two models with comparison to experimental evidences.

	*S. meliloti*		*R. etli*	
	*In vivo*	*iHZ565*	*In vivo*	*iOR363*
Complete citrate cycle	A [14], [35]	√	N [38]	√
Pentose phosphate pathway	A [14]	√	A [38]	×
Glyoxylate shunt	N [14], [39]	√	N.A.	N.A.
Poly-hydroxybutyrate cycle	N [28]	√	A [40]	√
Sulfur metabolism	A [8], [37]	√	A [16]	×
Porphyrin and chlorophyll metabolism	A [33]	√	A [33]	×

Abbreviation: A = active; N = inactive; √ = *in silico* simulation agrees with *in vivo* results; × = *in silico* simulation disagrees with *in vivo* results; N.A. = not available.

### Analysis of the reconstructed SNF metabolic network

Based on the reconstructed SNF metabolic network of *S. meliloti*, computational simulation and analysis of *i*HZ565 was performed by flux balance analysis (FBA). The flux distribution over the whole network was explored using the above defined OF under *in silico* microaerobic and nutrient sharing condition (see Methods). The predicted flux distribution of *i*HZ565 and its comparison to the reported experimental evidences, as well as comparison to the flux distribution of model *i*OR363, were listed in Table 2, which shows the solid consistency between model *i*HZ565 and the *in vivo* evidences. The comparison results were further elaborated as follows.

First, to make the process of bacteroid-host symbiotic nitrogen fixation on-going efficiently, tricarboxylic acid cycle (TCA), electron transfer chain, and nitrogen-fixing are required to be tightly coupled with each other, as shown in Figure 1. In *S. meliloti*, TCA started from the C_4_-dicarboxylic acids (*e.g.* malate or succinate), which was obtained from the host plants as the main carbon source to drive nitrogen fixation [3], [4], [35]. The *in silico* simulation showed that the oxidative TCA cycle was completely utilized in agreement with the reports in which all TCA enzymes (*i.e.* citrate synthase, aconitase, isocitrate dehydrogenase, 2-oxoglutarte dehydrogenase, succinyl-coa synthetase, succinic dehydrogenase, fumarase, and malate dehydrogenase) were detected in bacteroids [14], [35]. On the contrary, *i*OR363 predicted an incomplete utilization of TCA with the evidence of proteomic analysis of *Bradyrhizobium japonicum* bacteroids [38], in which all TCA enzymes are not always detected experimentally.For pentose phosphate pathway (PPP), the model *i*HZ565 predicted that PPP was active in agreement with the proteomic data of *S. meliloti*, in which several enzymes involved in the PPP were detected [14], [36]. The PPP is one of major pathways that in oxidative phase can generate NADPH, which is a reducing agent and essential for biosynthetic reactions that require reducing power (*e.g.* fatty acid synthesis). But the model *i*OR363 predicted that PPP in *R. etli* was inactive [17], contradicting the proteome data [38]. For pathway of Glyoxylate shunt, in model *i*HZ565, it was predicted to be inactive in *S. meliloti*, which agrees with the experimental data [14], [39]. The model *i*OR363 of *R. etli* did not provide indication of this pathway [17]. So far there is no *in vivo* result available. For poly-hydroxybutyrate (PHB) cycle, both *in vivo* and *in silico* results show that *S. meliloti* bacteroids do not accumulate PHB, which is a carbon storage compound [28], due to *S. meliloti* induces its legume hosts to form indeterminate nodules [28]; whereas *R. etli* can produce PHB as the consequence that its bacteroids induce the host plants to develop determinate nodules [40]. As for pathways of sulfur, porphyrin, and chlorophyll metabolism, they are especially vital to synthesis of nitrogenase and have been reported to be active in both *S. meliloti* and *R. etli* [8], [16], [33], [37]. The *in silico* simulation of *i*HZ565 shows the agreement with experimental data [33], while predictions by model *i*OR363 not [17]. All these characters and differences contribute directly to various underlying metabolic properties between *S. meliloti* and *R. etli.*

Besides the pathways listed in Table 2, computational simulation results also demonstrate that the gluconeogenesis pathway was active in *i*HZ565 and started from the TCA intermediate of malate, which converted to pyruvate catalysed by the NAD-malic enzyme (Dme, SMc00169) as the first step. The Dme and several common enzymes in gluconeogenesis in the nodule bacteria were detected [14]. It was also reported that Dme is required for SNF by bacteroids [41], as part of a pathway for the conversion of C_4_-dicarboxylic acids to acetyl-CoA. Consistent with this report, the simulation for Dme deletion in *i*HZ565 proved its inability to SNF. By computational simulation, amino acid cycling was also observed *in silico*. The model *i*HZ565 used glutamate to transaminate oxaloacetate or pyruvate to produce aspartate or alanine, respectively, and subsequently either or both of these amino acids were secreted. In the meantime, glutamate was obtained from the host plants. As the expectation, the nitrogen and carbon metabolism maintained the dynamic balance of equilibrium. When glutamate was the sole carbon and nitrogen source without any carbon source to provide to the *i*HZ565, the model maintained its proper functions including nitrogen fixation. On the other hand, when the carbon source was sufficiently provided, glutamate would be effused. The phenomenon evaluated the assumption that glutamate can be exported or imported to the bacteroids in order to regulate the balance between the carbon and nitrogen metabolism, with each using feedbacks to control the other [3]. In general, the carbon and nitrogen metabolism were intimately associated to support the efficient nitrogen fixation, as well as the mutual dependence between the bacteroids and host plants.

### Gap-filling and refinement of genome annotation

During the iterative process of metabolic reconstruction, gaps in the network were detected, leading to identification and refinement of improperly annotated genes of *S. meliloti* 1021. Totally, there were 15 genes whose annotations were refined via metabolic gap analysis, BLAST searches, and literature mining showed in Table 3. For instance, it was expected that cysteine should be synthesized, since it is one of important components required for the biosynthesis of glutathione and thioredoxin, which can maintain a reducing environment inside the cell (or bacteroids). However, the pathway of cysteine biosynthesis was interrupted by a reaction catalyzed by the enzyme - cystathionine beta-synthase (CBS), which has no gene assignment in current *S. meliloti* genome annotation. In addition, production of CBS was observed in the proteomic assay as well [14]. These evidences supported that the CBS should be catalyzed by certain gene. Therefore by BLAST searches, gene SMc04146 was identified with previous annotation of a “putative phosphoketolase” in the GenDB [42], and was reassigned the annotation of a “cystathionine beta-synthase”. These examples exemplified how reconstruction process can drive the refinement of gene functional annotation from different biological databases. Similar procedures were repeated for all of other model subsystems, until the model was able to generate a positive flux on objective function reaction while analyzing the model by FBA.

**Table 3:** Proposed annotation refinements.

EC	Gene name	Currentannotation	Proposed annotation	Reciprocal best hit#	*E*-value*
4.2.1.22	SMc04146	Putative phosphoketolase	Cystathionine beta-synthase	C6A884	0
4.1.1.15	SMa2402	RhsB L-2,4-diaminobutyrate decarboxylase	Glutamate decarboxylase	Q11XI9	1.00E-81
3.6.3.2	SMa1155	Cation transport P-type ATPase	Mg^2+^ importing ATPase	A9NGD8	3.00E-109
1.3.3.4	SMc00808	Putative chromate transport protein	Protoporphyrinogenase	D5BSU0	2.00E-78
1.2.1.70	SMc00727	Probable 3-hydroxybutyryl-CoA dehydrogenase	Glutamyl-tRNA reductase	B6KJQ6	8.00E-63
1.1.1.169	SM_b20362	Inositol-phosphate phosphatase	2-dehydropantoate 2-reductase	A8TQC8	3.00E-46
1.1.1.290	SMa2137	dehydrogenase	4-phosphoerythronate dehydrogenase	D4HBH5	3.00E-65
2.7.4.16	SMa0028	Selenophosphate synthase	Thiamine-phosphate kinase	E6PJ50	2.00E-13
3.1.3.15	SMa0483	Phosphatase	Histidinol-phosphatase	C5B1H3	6.00E-55
3.1.3.73	SMc04281	Probable threonine-phosphate decarboxylase	Cobalamin biosynthesis (CobC)	D4Z875	2.00E-37
3.1.3.74	SMc01617	Conserved hypothetical protein	Haloacid dehalogenase (HAD) superfamily hydrolase	C2GHB7	3.00E-12
3.2.2.8	SMc03175	Inosine-uridine preferring nucleoside hydrolase family protein	Ribosylpyrimidine nucleosidase	D3PL85	2.00E-43
1.4.3.10	SMc02377	Electron transfer flavoprotein-ubiquinone oxidoreductase	Putrescine oxidase	D5UT32	4.00E-08
3.1.2.14	SMc02273	Fatty acid synthase transmembrane protein	Acyl-[acyl-carrier-protein] hydrolase	B7K7V0	2.00E-135
N.A.	SMc01006	Hypothetical protein	NifU-like protein	B9JWF2	7.00E-50

# UniProt accession numbers.

*E-value based on reciprocal best hit against *S. meliloti* 1021 gene.

N.A. = not available.

### Analysis of the SNF essential genes

“SNF essential genes” are those that are indispensable for the efficient symbiotic nitrogen fixation in bacteroids. Determination of SNF essential genes provides a solid foundation for genomic experimentation and is helpful to understand the important functions required for nitrogen fixation to identify key genes in SNF. It is difficult to directly perform experiments in SNF stage and there are particular challenges in identification of SNF essential genes by large-scale knockout experimental approach. Taking into account the merits of *in silico* metabolic network analysis, which is complementary to the experimental deficiency, *i*HZ565 was used as a framework to predict the SNF essential genes in *S. meliloti* 1021 under the *in silico* microaerobic and nutrient sharing condition. About 19.8% (112) of the 565 metabolic genes included in *i*HZ565 were predicted to be essential, as listed in the File S3.

Unlike “essential genes”, which are those essential for an organism to survive under certain conditions (*e.g.* rich or minimal medium), “SNF essential genes”, are only crucial for the SNF stage, during which the bacteroids are non-dividing. These two terms concern two independent biological processes, yet it is expected that some fundamental functions might be shared and possibly, encoded by the homologous genes. The Database of Essential Genes (DEG) [43] has collected 12,297 experimental essential genes by now. It is possible to make comparative analysis between the SNF essential genes predicted by *i*HZ565 and the essential genes in DEG. The BLAST was performed against 15 prokaryotes in DEG with the criteria of *E*-value<1E-6 and identify >30%. Totally, there are 80 of the 112 predicted SNF essential genes that have homologs in DEG. Most of these genes are located on the biggest chromosome, which is in concordance with evidence from evolution. It has been reported that the chromosome was evolved precedent of the megaplasmids of pSymA and pSymB [6], which indicates that the chromosome contains almost all housekeeping genes. As the above evidences show that the functions encoded by these 80 genes, to some extent, are active not only in the SNF stage, but also in the developmental stages of bacteria. The high proportion of homologous genes indicates that the SNF requires the support of basic functions in independent processes.

**Figure 3:**
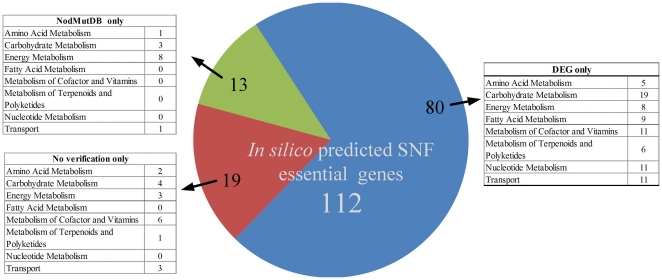
Comparison of *in silico* predicted SNF essential genes with genes from the Database of Essential Genes (DEG) and the Database of Nodule Mutants (NodMutDB).

Of the remaining 32 genes, the SNF essentiality of 13 genes can be validated by their corresponding mutants in previously published studies [28], [44]–[55], which have been collected in the Database of Nodule Mutants (NodMutDB). These mutants will cause Nod^+^Fix^−^ or Nod^+^Fix^+−^ phenotype, indicating that the bacteroids can form nodules but no or few nitrogen can be fixed. Comparison of *in silico* predicted SNF essential genes with genes from the Database of Essential Genes (DEG) and the Database of Nodule Mutants (NodMutDB) was showed in Figure 3. The rest 19 genes lack experimental evidences so far. Compared with other bacteria, these 19 genes showed their specificity for the SNF in *S. meliloti*, which can be further classified into two types of circumstances. First, since the genome of *S. meliloti* is not highly reiterated [8], some central functions are lack of alternative pathways. This makes some of these 19 genes essential. For example, pyruvate phosphate dikinase catalyzed in pyruvate metabolism are not essential in *E. coli*, as an alternative pathway exist and implement the same function. But its ortholog in *S. meliloti* (SMc00025) is indispensable in the SNF, because there's the lack of redundant pathways to facilitate the acquisition of new adaptive functions for symbiosis during *S. meliloti* evolution. Second, a number of genes are required for encoding nitrogenase-related cofactors (such as Fe-S, heme), which has an extremely important role in the SNF. These cofactors as fundamental compounds were included in the OF. An example is gene SMa0956, it encodes for glutamate-1-semialdehyde 2,1-aminomutase, which is a precursor for the biosynthesis of heme. These 19 genes in *S. meliloti* deserve further attention and experiments to validate their SNF essentiality in the future.

### Conclusions

In this study, we presented a manually curated a metabolic network of the SNF stage in *S. meliloti* 1021, which is known for its capability in symbiotic nitrogen fixation. An iterative reconstruction process led to generation of a reliable metabolic model, termed as *i*HZ565, which contains 503 metabolic, and transport reactions, 522 metabolites, 565 genes and all the main pathways participated in the stage of SNF. Besides this, biosynthetic pathways for some key cofactors required by nitrogenase, which is the only known family of enzymes accomplishing the process of atmospheric nitrogen gas fixation, were particularly emphasized and reconstructed, including heme, FeS cluster, molybdenum, and homocitrate.

To set an appropriate objective function that can accurately depict the specific biological process is a crucial step in the computational simulation and analysis of the reconstructed metabolic network. Here we gave a formulation as the OF which is distinguished from the one defined in *i*OR363, a previously published metabolic model for *R. etli* CFN42, which is also a nitrogen fixing bacterium but owns different physiological features from *S. meliloti*. In this study, the defined OF was based on the assumption that on the one hand, in the stage of SNF the biological process of nutrient sharing between plant and bacteroids should be tightly coupled with the process of nitrogen fixation in bacteroids; while on the other hand, the ammonia exported to plant should be generated by nitrogenase-catalysed reaction rather than other non-nitrogen fixation reaction indirectly generated. In order to balance these two biological processes and simultaneously optimize nitrogen fixation rate in the stage of SNF, we set both Reaction 1 and Reaction 2 as the OF in *i*HZ565. Subjected to this defined OF, the *in silico* predicted flux distribution was highly consistent with the *in vivo* evidences reported previously. Through an iterative process of the model reconstruction, the genome annotation of *S. meliloti* 1021 was further checked and 15 genes were properly re-annotated and listed in Table 3. Finally, the model *i*HZ565 was used as a complementary tool for the experimental deficiency to predict SNF essential genes. About 19.8% (112) of the 565 metabolic genes included in *i*HZ565 were predicted to be essential under the *in silico* microaerobic and nutrient sharing condition.

The metabolic reconstruction and refinement is a continuous process. The model *i*HZ565 will be further validated and improved with experimentally determined OF, large-scale gene deletion experimental data, proteomic data, and metabolomic data, as they become available for *S. meliloti*. Additionally, a SNF reconstruction of *S. meliloti* metabolism could propose several otherwise difficult research questions. For example, it would be informative to compare the *S. meliloti* metabolic network with metabolic networks of other related species. The comparison would allow us to investigate how these system-level properties might affect nitrogen fixation and would offer insight into mechanisms for nitrogen fixation and possible targets. In addition, *i*HZ565 can provides a valuable tool to explore the metabolic space of *S. meliloti*, describe its metabolic wiring under a range of conditions, pinpoint possible specific essential genes, generate testable hypotheses and improve nitrogen fixation in agriculture. In summary, our study underscored the value of the model *i*HZ565 as a framework to systematically study the SNF capabilities of *S. meliloti*.

## Materials and Methods

### Reconstruction of the SNF metabolic network

**Figure 4:**
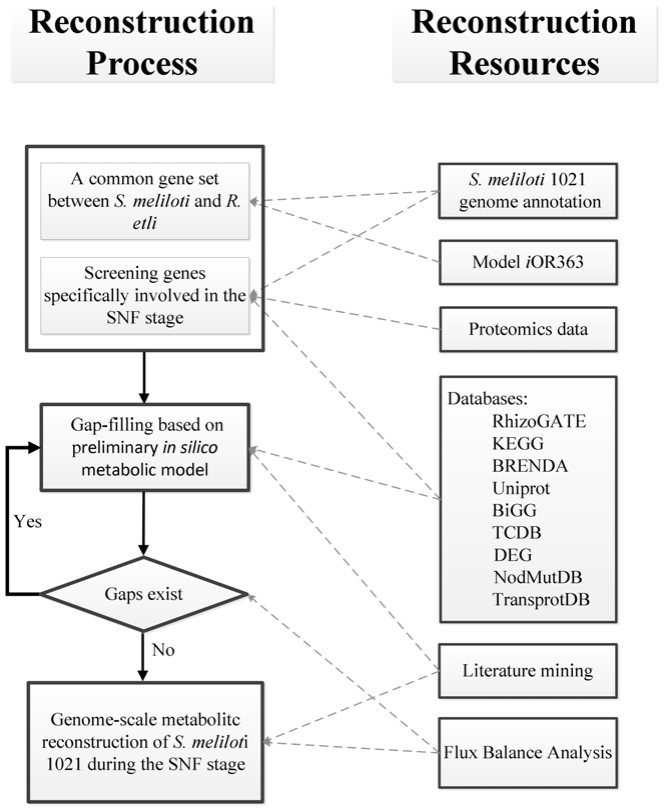
The process of metabolic reconstruction of the SNF stage in *S. meliloti* 1021. Resources used for the reconstruction are displayed on the right, and the reconstruction process is displayed on the left.

The metabolic reconstruction of *S. meliloti* 1021 focused on the SNF stage, during which the differentiated bacteroids are non-dividing and extremely distinguish from the normal bacterial cells. The reconstruction process for the symbiont, to some extent, has specific features as showed in Figure 4. Among the 6,292 annotated genes of *S. meliloti* 1021, only the genes expressed in the SNF stage were selected. For the initial reconstruction, one part of genes was collected from the putative orthologs between the annotated genes of *S. meliloti* 1021 and the genes of *R. etli* CFN42 incorporated in the *i*OR363. Considering that both *S. meliloti* and *R. etli* are α-proteobacteria symbiotic nitrogen fixation bacteria, it was expected that many orthologs would exist,and hence have shared functions. As a result, there were 312 homologous genes identified through finding highly homologous gene pairs, known as “reciprocal best genes (RBGs)” [56], by using BLAST algorithm against the *S. meliloti* 1021 genome sequence with *i*OR363-contained genes as quires and vice versa. The searches were performed using the criteria of *E*-value<1E-6, identify >30%, and matched length >60%. The other part of genes was screened according to proteomic data [14], which determined the enzymatic reactions in the *S. meliloti* when it occupies the root nodules. Totally, there were 73 reported genes can be directly incorporated into the initial reconstruction with high confidence. These selected genes were subsequently associated with corresponding proteins and reactions to establish the association of gene-protein-reaction (GPR). Following previous work [57], we represented GPR associations by Boolean logic statements, which interlink genes with protein complexes and protein with reactions by the combinations of “AND” or “OR” operators. An “AND” operator indicates that two or more genes are required to encode a protein as in the case of multi-protein complexes, while an “OR” operator indicates that any of several genes can encode a protein as in the case of isozymes. The reversibility of each reaction was determined based on literature sources, BRENDA [58], and BiGG [59] databases, when available or based on simple thermodynamic considerations [60]. The initial reconstruction was not a completed network where existed some gaps that failed to perform flux balance analysis. An iteratively process of gap-filling and model improvement is necessary by using BLAST similarity searches between the translated set of *S. meliloti* and enzymes from public databases with the annotation of interest. During the metabolic reconstruction of *S. meliloti*, various biological databases were used, including KEGG [61], NCBI [62], GeneDB [42], UniProt [63], BRENDA [58], BiGG [59], NodMutDB [64], DEG [43], RhizoGATE [65], Transport Classification Database (TCDB) [66], and TransportDB [67]. The comprehensive map of metabolic reconstruction of *S. meliloti* 1021 in the SNF stage was available in the File S1.

### Flux balance analysis

Flux balance analysis (FBA) is one of the widely used constraint-based methods in computational analysis of the reconstructed metabolic networks. Based on the assumption of a pseudo-steady state, FBA applies an optimal algorithm of linear programming (LP) to search the best flux distribution subjected to an optimized objective function within a bounded solution space, which is defined in terms of mass balance constraints imposed by thermodynamics (*i.e.* the reaction is reversible or not) and enzyme or transporter capacities (*i.e.* the maximum uptake or the limits of reaction rates), as described previously [18]–[20]. Optimization of the defined OF and simulation of single gene deletion were calculated using the Constraint-based Reconstruction and Analysis (COBRA) [68] Toolbox which is a set of MATLAB scripts for constraint–based modelling that are run from within the MATLAB environment.

### *In silico* simulation of nutrient sharing system

The symbiont depends on nutrient sharing system to ensure the continuous nitrogen fixation. To *in silico* simulate the symbiont habitat, either uptake or excretion of the extracellular metabolites is via exchange reactions. Specifically, malate (or succinate) as a major carbon and energy source was obtained from the plant with the uptake rate of 1.112 mmol/gDW/hr (succinate is 1.326 mmol/gDW/hr) [69]. Glutamate was also obtained from the plant and in return, the synthesized alanine and aspartate in bacteroids were excreted back to the plant, which played an important role in the amino acid cycling between the plant and bacteroids [35]. As reported, microaerobic environment is necessary for SNF, the oxygen uptake rate was limited to 1.26 mmol/gDW/hr [70]. Other exchanged compounds included iron, sulfate, inositol, phosphate, homocitrate, magnesium, hydrogen, carbon dioxide, and water [14], [32], [71]–[74]. Furthermore, the inositol sink was limited the uptake rate of 0.01 mmol/gDW/hr because the inositol was observed in bacteroids, but it is unknown whether it is supplied by the plant or synthesized inside the bacteroids. The detailed constraints on each of reaction are listed in File S1.

## Supporting Information

File S1 **The reconstructed SNF metabolic model** ***i*** **HZ565.**
(XLS)

File S2 **The model** ***i*** **HZ565 in SBML format.**
(XML)

File S3 ***In silico*** **predicted SNF essential genes and comparison results.**
(XLS)
